# Perceived patient safety culture and its associated factors among clinical managers of tertiary hospitals: a cross-sectional survey

**DOI:** 10.1186/s12912-023-01494-4

**Published:** 2023-09-25

**Authors:** Haiyan He, Xi Chen, Lingyun Tian, Yanfang Long, Li Li, Ning Yang, Siyuan Tang

**Affiliations:** 1 Teaching and Research Section of Clinical Nursing, Xiangya Hospital, Central South University, 87 Xiangya Road, Changsha, 410008 Hunan China; 2 Xiangya School of Nursing, Central South University, Changsha, Hunan China; 3 School of Nursing, Hong Kong Polytechnic University, Hongkong, China; 4 Department of Nursing, The First Affiliated Hospital of USTC, Division of Life Sciences and Medicine, University of Science and Technology of China, Hefei, Anhui China; 5 Emergency Department, Xiangya Hospital, Central South University, Changsha, Hunan China

**Keywords:** Adverse events reported, Associated factor, Clinical manager, Patient safety culture, Patient safety grade, Time delays per shift, Tertiary hospital

## Abstract

**Background:**

Patient safety is a global challenge influenced by perceived patient safety culture. However, limited knowledge exists regarding the patient safety culture perceived by hospital clinical managers and its associated factors. This study aims to investigate the perceptions of patient safety culture and associated factors among clinical managers of tertiary hospitals in China.

**Methods:**

A cross-sectional survey was conducted from June 19 to July 16, 2021, involving 539 clinical managers from four tertiary hospitals in Changsha City of Hunan Province. The Hospital Survey on Patient Safety Culture (HSOPSC) was utilized to assess perceived patient safety culture. Bivariate, multivariable linear regression, and logistic regression analyses were performed.

**Results:**

The mean score for the total HSOPSC was 72.5 ± 7.6, with dimensional scores ranging from 62.1 (14.9) to 86.6 (11.7). Three dimensions exhibited positive response rates (PRRs) < 50%, indicating areas that need to be improved: “nonpunitive response to errors” (40.5%), “staffing” (41.9%), and “frequency of events reported” (47.4%). Specialized hospitals (β = 1.744, *P* = 0.037), female gender (β = 2.496, *P* = 0.003), higher professional title (β = 1.413, *P* = 0.049), a higher education level (β = 1.316, *P* = 0.001), and shorter time delays per shift (β=-1.13, *P* < 0.001) were correlated with higher perceived patient safety culture. Education level, work department, “teamwork within a unit”, “management support for patient safety”, “communication openness”, and “staffing” dimensions were associated with patient safety grades (all *P* < 0.05). Years worked in hospitals, occupation, education level, work department, hospital nature, professional title, “communication openness”, and “handoffs & transitions” were associated with the number of adverse events reported (all *P* < 0.05).

**Conclusions:**

Our study revealed a generally low level of patient safety culture perceived by clinical managers and identified priority areas requiring urgent improvement. The associated factors of patient safety culture provide important guidance for the development of targeted interventions in the future. Promoting patient safety by optimizing the patient safety culture perceived by clinical managers should be prioritized.

**Supplementary Information:**

The online version contains supplementary material available at 10.1186/s12912-023-01494-4.

## Background

Patient safety is a cornerstone within the evolving healthcare landscape [1]. Safeguarding patient well-being in the current dynamic healthcare environment stands as a significant challenge [2]. Alarmingly, approximately one in ten hospitalized patients worldwide experience preventable safety failures during their treatment [3]. Medical staff are frequently exposed to workplace violence, often stemming from a lack of awareness regarding patient rights [4, 5]. Patient safety, recognized as a basic patient right, has evolved into a fundamental requirement for hospital accreditation, reflecting its pivotal role in healthcare delivery [6].

Patient safety culture comprises a multidimensional framework that includes shared values, beliefs, attitudes, and behaviors to promote patient safety and minimize harm [7]. It encompasses strategies to prevent patient harm, underscores the significance of error prevention and learning from mistakes, and contributes to the establishment of a robust healthcare system [2]. Conversely, inadequate perceptions of patient safety culture are associated with high rates of medical errors and adverse events in healthcare [8, 9]. Fostering a robust patient safety culture becomes imperative in preventing adverse events, improving the quality of care, and safeguarding patient safety within healthcare systems [10–12].

An accurate assessment of patient safety culture is crucial for understanding health professionals’ perceptions and prioritizing interventions [13]. The Hospital Survey on Patient Safety Culture (HSOPSC) is widely used and validated for evaluating patient safety culture across different countries [14, 15]. Developed by Westat and released by the Agency for Healthcare Research and Quality [16], the HSOPSC has been translated into 30 + languages and used in 60 + countries, showing strong psychometric properties [15]. International studies strongly suggest a global adoption of HSOPSC, with notable utilization in the United States, Europe, and Asia [15]. Safety culture in Asian countries varies due to lower prioritization of patient safety policy compared to developed nations [17]. Healthcare systems should prioritize consolidating patient safety culture at all levels in line with health policy to achieve sustainable development goals [17].

Although numerous studies have examined patient safety culture using the HSOPSC, most of these studies have primarily focused on the perceptions of front-line nurses [18]. While the role of healthcare providers in delivering care and ensuring patient safety is widely acknowledged, it is equally important to recognize the significance of clinical managers in fostering a culture of safety at the organizational level [19]. Clinical managers play a vital role in fostering a positive patient safety culture and creating nonpunitive environments to promote patient safety [20, 21]. Their leadership and managerial activities significantly influence medical staff adherence to hospital processes, particularly in areas such as teamwork and communication [22]. Moreover, clinical managers’ perceptions of patient safety culture have a substantial impact on various aspects, including error reporting [23], the care process [22], relational quality [19], and patient satisfaction [24].

Several studies have demonstrated that hospital clinical managers hold distinct perceptions of patient safety culture compared to other healthcare providers [24–26]. The safety attitudes of clinical managers were generally poor and required improvement [27, 28]. Studies have identified three influential factors on patient safety culture: sociodemographics, work-related factors, and organizational factors [29, 30]. However, few studies have explored the factors shaping managers’ perceptions [27, 28]. Zhang et al. conducted a study exploring the correlation between safety attitudes and safety factors among clinical managers utilizing the Safety Attitudes Questionnaire [27]. There is a notable gap regarding research on clinical managers’ perceptions concerning patient safety culture [27]. Abraham et al. examined patient safety culture as perceived by managerial staff in a tertiary hospital in South Africa through qualitative interviews, with a primary focus on identifying areas requiring improvement [28].

A theoretical framework for safety culture comprises patient safety culture dimensions, influencing factors, and interventions for enhancement [31]. It emphasizes essential components: effective communication, organizational commitment to learning, transparent reporting, teamwork, and managerial support [31]. Notably, safety culture is shaped by both internal and external factors. This framework is designed to guide stakeholders in devising strategic plans to bolster safety culture and, consequently, advance patient safety [31].

Recognizing the crucial role of clinical managers in cultivating a patient safety culture is imperative. However, there is a poor level of safety attitudes among clinical managers, and a deeper exploration of the factors influencing their perceptions is warranted [27, 32]. To address this research gap, it is crucial to examine the perceived patient safety culture specifically among clinical managers in tertiary hospitals in China. This study aims to explore perceptions of patient safety culture and identify their influential factors, which are practical in informing strategies to enhance patient safety in healthcare organizations. The findings offer insights for targeted strategies to improve patient safety culture and overall healthcare quality.

## Methods

### Study design

A cross-sectional survey was conducted in Changsha, Hunan Province, China, from June 19 to July 16, 2021. The study employed the HSOPSC and utilized a multistage random sampling method.

### Setting and sample

Changsha, the capital city of Hunan Province, is located in central China and has an annual per capita income of $8,000. The city is home to 29 tertiary state-owned hospitals [33]. Eligible participants were hospital clinical managers, including physicians’ directors and head nurses working in different clinical departments of these hospitals.

Inclusion criteria required participants to be clinical managers aged 20 to 60 years with at least one year of experience in their managerial role and to have received patient safety culture training in hospitals. Exclusion criteria encompassed clinical managers who were no longer on duty or not currently working in the hospital due to reasons such as overseas assignments, illness, or maternity leave.

The sample size was calculated based on a power of 0.80, an alpha of 0.05, and an allowable error of 0.001. The calculation was performed using the mean and standard deviation of the “overall patient safety grade” among managers (4.0 ± 1.0) obtained from a previous study [32]. Taking into account a potential nonresponse rate of 10–20%, a final sample size of 560 participants was determined [34].

A multistage random sampling method was used to account for variations in perceptions of a patient safety culture based on geographic regions and hospital scale. (Supplementary Fig. 1). Two areas (east and west) were randomly selected from Changsha City, and from each area, one large hospital (with more than 2000 beds) and one small hospital (with fewer than 2000 beds) were chosen. Finally, four hospitals were selected, including a general hospital, a maternal and child health hospital, an oncology hospital, and a tuberculosis hospital. All clinical managers from these hospitals were invited to participate, resulting in a total distribution of 560 questionnaires with a response rate of 98.9%. After removing 15 repetitive questionnaires, 539 valid questionnaires were included in the analysis.

### Instruments

#### Participant information

Demographic and background information was collected, including gender, age, educational level, marital status, occupation, professional title, form of employment, work department, working years in hospitals, time delays per shift, number of night shifts per month, direct contact with patients, hospital scale and hospital nature.

#### Hospital survey on patient safety culture (HSOPSC)

The HSOPSC, developed by Westat and endorsed by the Agency for Healthcare Research and Quality, serves as a tool for evaluating the perceived patient safety culture in hospitals [16]. It demonstrates strong psychometric attributes that enhance its reliability and validity [15]. It consisted of 42 items grouped into 12 dimensions, including “teamwork within units”, “supervisor/manager expectations and actions promoting patient safety”, “organizational learning and continuous improvement”, “management support for patient safety”, “overall perceptions of patient safety”, “feedback and communication about errors”, “communication openness”, “frequency of events reported”, “teamwork across units”, “staffing”, “handoffs and transitions”, and “nonpunitive response to errors” dimensions [15]. Each item was rated on a five-point Likert scale from 1= “strongly disagree” to 5= “strongly agree” for agreement or from 1= “never” to 5= “always” for frequency. Eighteen negatively worded items were reverse-scored. The linearly converted scores of each dimension or item ranged between 0 and 100 [32], with higher scores indicating a stronger patient safety culture [16]. To determine the strength of each item or dimension, a positive response rate (PRR) was calculated based on responses of “strongly agree/agree” or “always/most of the time”. PRRs above 75% were considered strengths, while those below 50% indicated areas for improvement [16]. Additionally, two items were added to measure the level of patient safety and the number of adverse events reported over the past 12 months.

This survey questionnaire was constructed based on the HSOPSC and was collaboratively developed with the expertise of seven specialists in hospital safety management [35]. The questionnaire was adapted to align with the specific conditions of the local healthcare context. Furthermore, a pilot study involving 20 managers was conducted to assess the questionnaire’s face validity and clarity [35]. The total HSPSC showed an acceptable Cronbach’s α coefficient of 0.88, and the Cronbach’s α coefficient of each dimension ranged from 0.88 to 0.89 [35].

#### Data collection

All eligible clinical managers were recruited to participate in the study via a prenotification email sent to hospital managers. The permissions for the survey participants were obtained from them during patient safety training among managers. Data were collected using an online survey tool called Wenjuanxing (https://www.wjx.cn). Participants received a survey link through WeChat (the primary means of mobile communication in China) to increase response rates. Clear instructions were provided at the beginning of the questionnaires to ensure data integrity and accuracy. To minimize missing values, the questionnaire was designed with a function that reminded respondents to answer any unanswered questions before submitting the survey. Participants completed the questionnaires voluntarily, indicating their informed consent. Questionnaires with identical responses for each item in sections A, B, C, and F were excluded because these sections contain both positively and negatively worded items [16]. Two researchers independently recorded and verified the collected questionnaires.

### Statistical analysis

Categorical variables are presented as frequencies and percentages; continuous data are reported as the mean (M) and standard deviation (SD). Data were checked for normality using Kolmogorov–Smirnov testing. PRRs were defined as the proportion of positive responses for each dimension or item. Independent *t* tests, one-way analysis of variance, or Welch analysis of variance were used for group comparisons. Additionally, comparisons were made between the total scores of the HSOPSC and its dimensions based on time delays per shift. Multivariate linear regression analysis was performed, treating demographic and background variables as independent variables and the total score of the HSOPSC as the dependent variable. Dummy variables were used to represent demographic and background variables, and a forward LR approach was employed.

Bivariate and multiple logistic regression analyses were conducted to examine the relationship between the outcome variables (number of adverse events reported and overall patient safety grade) and the explanatory variables (sociodemographic variables and 10 dimensions of patient safety culture). The outcome variable “overall patient safety grade” was dichotomized into positive (i.e., “excellent” and “very good”) and negative (i.e., “failing” to “acceptable”). The variable “number of events reported” was dichotomized as “no event reports” and “one event report or more”. A two-sided *p* value of less than 0.05 represented statistical significance. Data analysis was performed using SPSS version 28.

### Ethical considerations

The study was approved by the ethical committee of Xiangya Hospital of Central South University (202,011,159). Informed consent to participate in the research was received from clinical managers in the study. Participants were also guaranteed the personal anonymity and confidentiality of the data. Participants were also assured of individual anonymity and confidentiality of data without the use of individual identifiers. The researchers clearly stated the objectives, benefits, and potential risks to participants. They guaranteed the right of participants to withdraw from the study. Data were secure and accessible only to researchers. They were also responsible for data management and data storage.

## Results

### Participant characteristics

Table 1 presents the characteristics of the 539 clinical managers from four tertiary hospitals in Changsha City, Hunan. In terms of sociodemographics, the majority of participants were female (74.6%), married (87.8%), and aged between 30 and 50 (76.2%). Regarding educational background, 53.6% had a bachelor’s degree or below. In terms of work-related characteristics, most participants were nurses (62.6%), employed as agency staff (86.6%), and held a junior professional title (51.0%). They worked in various departments, including internal medicine (30.0%), surgery (27.3%), and other departments (42.7%). The majority of participants worked in specialized hospitals (73.8%), had over 10 years of work experience (69.2%), and had direct contact with patients (84.2%). More than half of them had at least one night shift per month (56.4%), and the majority experienced delays of at least half an hour per shift (67.7%). Significant differences in HSOPSC scores were observed based on gender (*P* < 0.05) and time delays per shift (*P* < 0.001).

**Table 1:** Participant characteristics and mean scores of the HSOPSC (n = 539)

Variables	N (%)	Mean (SD)	*p*
**Gender**			0.005*
Male	137(25.4)	70.9(7.8)	
Female	402(74.6)	73.0(7.5)	
**Age (years)**			0.131
20~29	53(9.8)	72.7(7.8)	
30~39	211(39.2)	72.3(8.0)	
40~49	200(37.1)	73.3(7.1)	
50~60	75(13.9)	70.9(7.5)	
**Educational level**			0.280
Bachelor’s degree and below	289(53.6)	72.2(7.0)	
Master and above	250(46.4)	72.9(8.3)	
**Marital status**			0.994
Married	473(87.8)	72.5(7.7)	
Single	66(12.2)	72.5(7.2)	
**Occupation**			0.126
Nurse	335(62.2)	72.9(7.3)	
Doctor	204(37.8)	71.8(8.1)	
**Professional title**			0.356
Junior	275(51.0)	72.2(7.3)	
Senior	264(49.0)	72.8(8.0)	
**Form of employment**			0.350
Agency staff	467(86.6)	72.4(7.6)	
Contract staff	72(13.4)	73.3(8.0)	
**Work department**			0.115
Internal medicine department	162(30.0)	73.6(7.9)	
Surgery department	147(27.3)	72.2(6.6)	
Others	230(42.7)	72.0(8.1)	
**Working years in hospitals**			0.942
1~10	166(30.8)	72.5(8.0)	
11~20	164(30.4)	72.3(8.3)	
> 20	209(38.8)	72.6(6.8)	
**Time delays per shift (hours)**			< 0.001**
< 0.5	174(32.3)	74.6(7.5)	
0.5~1.5	216(40.1)	72.3(6.8)	
> 1.5	149(27.6)	70.3(8.4)	
**Number of night shifts per month**			0.834
0	235(43.6)	72.7(7.2)	
1~4	140(26.0)	72.2(7.9)	
> 4	164(30.4)	72.5(8.1)	
**Direct contact with patients**			0.921
Yes	454(84.2)	72.5(7.5)	
No	85(15.8)	72.4(8.4)	
**Hospital scale (beds)**			0.162
< 1000	161(29.9)	73.2(7.1)	
1000~2000	237(44.0)	72.5(8.0)	
> 2000	141(26.1)	71.6(7.6)	
**Hospital nature**			0.092
General Hospital	141(26.2)	71.6(7.6)	
Specialized hospital	398(73.8)	72.8(7.6)	

HSOPSC: Hospital Survey on Patient Safety Culture; SD, standard deviation

*P < 0.05; **P < 0.001

### Total score and PRRs of the HSOPSC

Table 2 presents the total scores for the HSOPSC and its dimensions categorized by time delays per shift. The mean total score of the HSOPSC was 72.5 ± 7.6, while the dimensional scores ranged from 62.1 (14.9) to 86.6 (11.7). The dimensions with the highest mean scores were “teamwork within units” (M = 86.6, SD = 11.7), “organizational learning—continuous improvement” (M = 84.4, SD = 10.2), and “feedback & communication about error” (M = 80.2, SD = 12.2). Additionally, the dimensions with the lowest mean scores were “nonpunitive response to errors” (M = 62.1, SD = 14.9), “staffing” (M = 63.5, SD = 14.8), and “frequency of events reported” (M = 68.3, SD = 16.9). Apart from the “frequency of events reported” and “handoffs & transitions” dimensions, the HSOPSC and its other dimensions exhibited statistically significant differences in time delays per shift (all *P* < 0.05).

Supplementary Table 1 provides the mean scores and the PRRs for each dimension or item within the HSOPSC among hospital managers, comparing PRRs in China and the USA based on the 2021 Hospital 1.0 Database. China had lower PRRs for most items compared to the USA, except for higher PRRs in the “organizational learning & continuous improvement” and “handoffs & transitions” dimensions. The PRR for the total HSOPSC was 63.9%, while the dimensional PRRs ranged from 40.5% to 90.2%. Notably, the dimensions of “teamwork within units” (90.2%), “organizational learning and continuous improvement” (89.5%), “feedback and communication about errors” (77.6%), and “supervisor/manager expectations and actions promoting patient safety” (75.7%) had PRRs above 75%, indicating strengths. On the other hand, the dimensions of “nonpunitive response to errors” (40.5%), “staffing” (41.9%), and “frequency of events reported” (47.4%) had PRRs below 50%, highlighting areas that need improvement. Among the 42 items, the top three items with the highest PRRs were “F3. Things ‘fall between the cracks’ when transferring patients from one unit to another” (89.35%), “A1. People support one another in this unit” (91.34%), and “A6. We are actively doing things to improve patient safety” (94.4%). In contrast, the top three items with the lowest PRRs were “A16. Staff worry that mistakes they make are kept in their personnel file” (12.82%)”, “A14. We work in ‘crisis mode’ trying to do too much, too quickly” (28.16%) and “A5. Staff in this unit work longer hours than is best for patient care” (30.32%) [16].

**Table 2:** Mean scores of the HSOPSC and its dimensions by time delays per shift

	Time delays per shift (hours)				*p*
	<=0.5	0.5–1.5	>=1.5	Total	
Teamwork Within Units	88.9(10.3)	86.9(10.8)	83.7(13.8)	86.6(11.7)	0.001*
Supervisor/Manager Expectations & Actions Promoting Patient Safety	80.6(12.2)	78.8(11.6)	74.9(12.6)	78.3(12.2)	< 0.001**
Organizational Learning and Continuous Improvement	86.5(9.3)	83.8(9.5)	82.6(11.7)	84.3(10.2)	0.001*
Management Support for Patient Safety	81.9(12.3)	76.8(13.4)	73.2(14.7)	77.5(13.8)	< 0.001**
Overall Perceptions of Patient Safety	76.5(11.8)	72.0(11.4)	71.0(14.0)	73.2(12.5)	< 0.001**
Feedback & Communication About Error	82.8(11.8)	80.0(11.4)	77.5(13.0)	80.2(12.2)	0.001**
Communication Openness	73.9(12.3)	71.2(11.6)	69.5(13.2)	71.6(12.4)	0.005*
Frequency of Events Reported	68.4(18.9)	68.8(15.6)	67.5(16.4)	68.3(16.9)	0.750
Teamwork Across Units	74.6(11.9)	72.1(11.2)	70.4(12.9)	72.4(12.0)	0.006*
Staffing	68.9(14.3)	62.9(14.1)	58.2(14.2)	63.5(14.8)	< 0.001**
Handoffs & Transitions	70.0(13.9)	68.3(12.1)	67.5(13.8)	68.6(13.2)	0.189
Nonpunitive Response to Errors	65.3(14.6)	61.9(14.9)	58.7(14.5)	62.1(14.9)	< 0.001**
The total HSOPSC	74.6(7.5)	72.3(6.8)	70.3(8.4)	72.5(7.6)	< 0.001**

HSOPSC: Hospital Survey on Patient Safety Culture. *P < 0.05; **P < 0.001

### Factors associated with patient safety culture

Table 3 presents the results of the multivariate regression analysis after controlling for all demographic and background variables, indicating the factors associated with perceived patient safety culture. Five factors remained statistically significant: hospital nature (β = 1.744, *P* = 0.037), gender (β = 2.496, *P* = 0.003), professional title (β = 1.413, *P* = 0.049), education level (β = 1.316, *P* = 0.001), and time delays per shift (β=-1.13 *P* < 0.001).

**Table 3:** **Multivariate regression analysis of the HSOPSC (n = 539)**

Variables	*B*	*S*_*b*_	*Beta*	*t*	*p*
Constant	62.335	3.232		19.285	< 0.001**
Time delays per shift	-1.13	0.214	-0.229	-5.275	< 0.001**
Education level	1.316	0.396	0.172	3.320	0.001*
Gender	2.496	0.823	0.142	3.034	0.003*
Professional title	1.413	0.716	0.092	1.973	0.049*
Hospital nature	1.744	0.834	0.100	2.091	0.037*

*B*: unstandardized regression coefficient; *Beta*: standardized regression coefficient

*P < 0.05; **P < 0.001

**Fig. 1:**
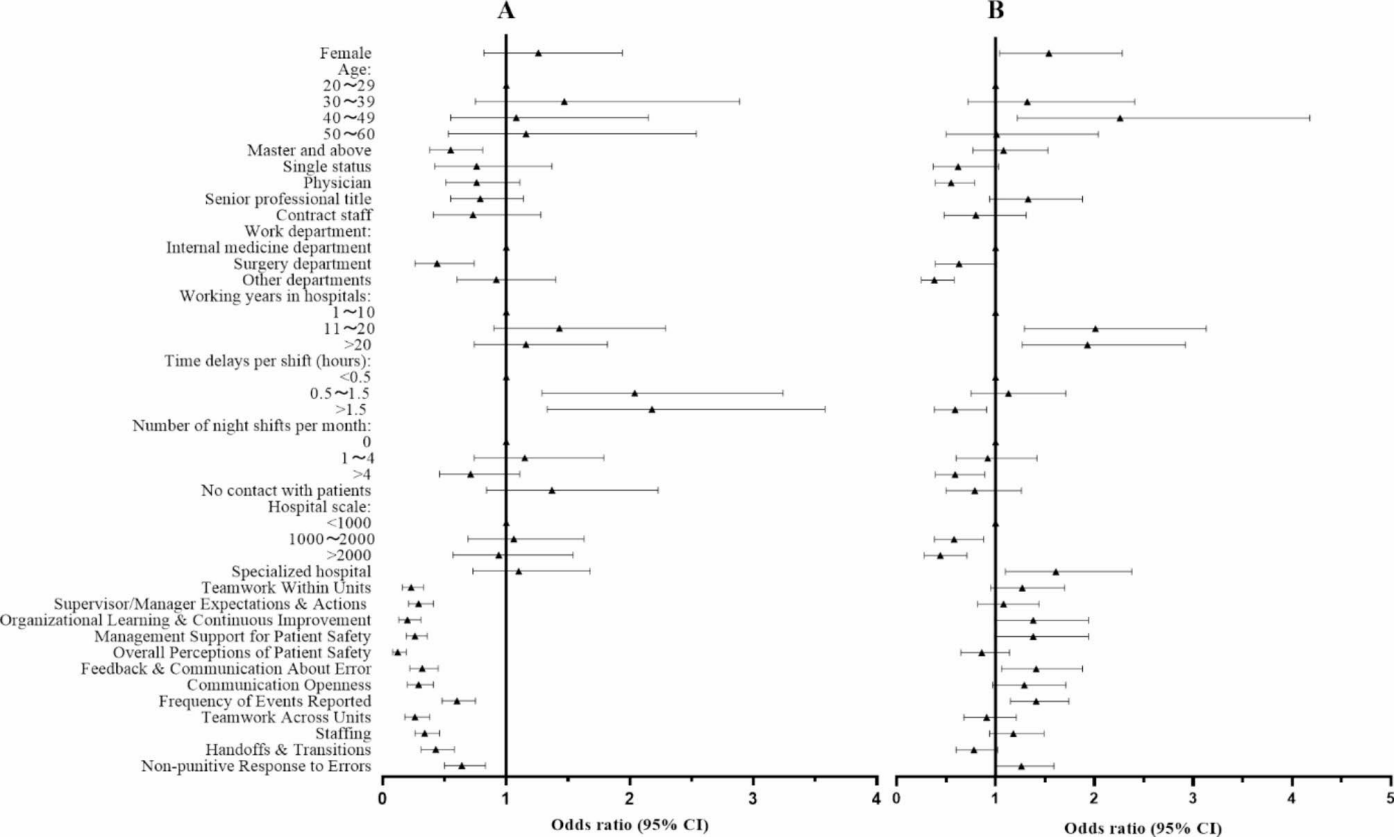
Binary logistic regression analysis for **(A)** patient safety grade and **(B)** number of adverse events reported

A total of 374 participants (69.4%) reported their patient safety grade as excellent/very good, while 155 participants (28.7%) rated it as acceptable, and only 10 participants (1.9%) perceived it as poor/failing. Regarding adverse events reported over the past 12 months, 221 participants (41.0%) did not report any events, 189 participants (35.1%) reported 1 to 2 events, and 129 participants (23.9%) reported 2 events or more. Figure 1 presents the binary logistic regression analysis results, examining the relationship between participant characteristics, HSOPSC dimensions, patient safety grade (Fig. 1A), and the number of adverse events reported (Fig. 1B).

**Fig. 2:**
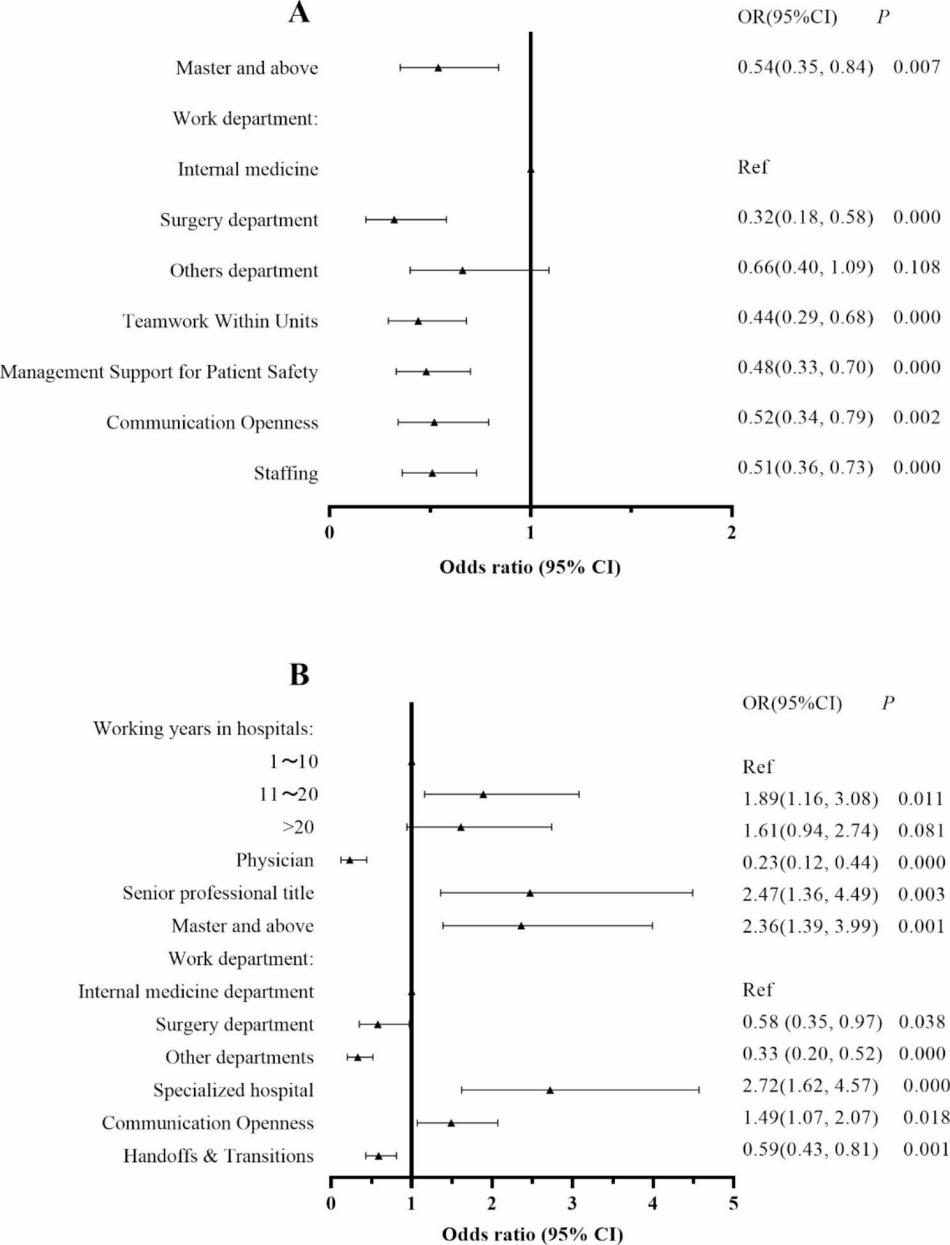
Multiple logistic regression analysis for **(A)** patient safety grade and **(B)** the number of adverse events reported

The results of the multiple logistic regression analysis are summarized in Fig. 2. The internal medicine department participants were more likely to perceive higher patient safety grades than those in the surgery department. Respondents with a bachelor’s degree and below were also more likely to perceive better patient safety grades than those with a master’s degree and above. Moreover, an increase of one unit in the scores for the “teamwork within a unit”, “management support for patient safety”, “communication openness”, and “staffing” dimensions was associated with higher odds of reporting a positive patient safety culture (Fig. 2A).

The odds of reporting adverse events were found to be higher among participants with a higher education level, higher professional titles, and longer working years in hospitals. Additionally, nurses were more likely to report events than physicians. The internal medicine department participants had higher odds of reporting adverse events than those from the surgery department and other departments. Clinical managers in specialized hospitals also had higher odds of reporting adverse events than those in general hospitals. Furthermore, an increase of one unit in the scores for the “communication openness” and “handoffs & transitions” dimensions was associated with higher odds of reporting adverse events (Fig. 2B).

## Discussion

In the current study, we investigated the perception of patient safety culture and its associated factors among clinical managers in Central China. To our knowledge, no previous cross-sectional study has examined the influencing factors of patient safety culture among clinical managers. Overall, the scores for both the total HSOPSC and its dimensions were relatively low compared to the results of other studies [26, 32]. Three strength areas were identified and should be maintained. Three dimensions, including “nonpunitive response to errors”, “staffing”, and “frequency of events reported”, had the lowest mean scores and PRRs, highlighting areas that require improvement. Factors influencing patient safety culture included specialized hospitals, gender (females), higher professional titles, higher education levels, and shorter time delays per shift. The association of participant characteristics and HSOPSC dimensions with patient safety grade and the number of reported adverse events were also examined.

The “nonpunitive response to errors” dimension exhibited the lowest mean score and PRR, indicating a pressing need for improvement. Clinical managers worried that mistakes they made would be kept in their records and affect their future career development in this study. This finding aligns with a recent literature review, which revealed that the dimension of “nonpunitive response to errors” was weak in most of the included studies [36]. The majority of frontline staff expressed the lowest perceptions of the “nonpunitive response to errors” dimension, which was aligned with other Asian countries [37, 38]. Ineffective leadership and a culture of blame were identified as key factors impeding the development of a positive patient safety culture [38]. The presence of a punitive atmosphere emerged as the primary barrier hindering their willingness to report adverse events, identify potential causes, and facilitate learning from errors [10]. A blame-oriented culture has significant implications for healthcare quality and poses a threat to patient safety [39]. Our study highlighted the critical importance of hospitals prioritizing the establishment of a blame-free culture and providing a nonpunitive response to staff errors. This approach facilitated proactive risk assessment and enhanced hospitals’ capacity to respond to incidents using training and the execution of discussion and operation-based exercises [40, 41]. Another potential explanation related to the negatively worded items within the “nonpunitive response to errors” dimension may introduce a comprehensiveness issue due to the increased difficulty in understanding negatively worded questions compared to positively worded ones [42]. The lowest score observed in this dimension may reflect a limitation in the wording rather than an accurate reflection of the actual culture [36]. Therefore, it is necessary to conduct further research on scale modification and psychometric testing to enhance its validity.

The “staffing” dimension is also a crucial area that requires improvement, supported by findings from the HSOPSC Comparative Database in the USA [43] and European countries [15]. Both items with the lowest scores in the HSOPSC pertain to staffing, underscoring the critical nature of the staffing issue. This finding aligns with consistent reports of low PRRs in the staffing dimension across various studies [7, 15, 38]. In our study, clinical managers frequently worked night shifts and experienced time delays per shift. These circumstances, coupled with overwhelming workloads and extended hours, contribute to high levels of burnout and adversely impact the quality of care, posing potential risks to patient safety [22, 27]. In our study, longer time delays per shift were the most significant factor associated with a negative patient safety culture, as evidenced by the relationship between self-reported workload and perceptions of patient safety culture [44]. Additionally, time delays per shift were linked to overall patient safety grades and the number of reported adverse events in the bivariate logistic regression analysis. As previously discussed, increased time delays per shift reflect higher workloads, which may lead to staff burnout and compromised patient safety [22, 27]. These findings emphasize the need for increased staff support and reduced workload to improve patient safety culture and enhance the quality of care.

The “frequency of events reported” dimension is another area of concern that requires improvement. Our study found that nearly half of the clinical managers did not report any adverse events in the past year, suggesting a punitive patient safety culture that discourages active reporting by clinical managers [39]. Clinical managers in our study exhibited lower perception levels compared to a prior research study that evaluated and compared patient safety culture among healthcare providers in Chinese hospitals [32]. A multinational study demonstrated that the frequency of events was predominantly impacted by feedback and communication [45]. To enhance patient safety, there is a need to prioritize and improve communication practices, particularly in the context of error reporting, as indicated by this study comparing patient safety culture in diverse cultural settings [45]. The significance of patient safety culture must be acknowledged among both clinical managers and staff to foster teamwork and communication, enhancing organizational culture and practices [22]. These findings underscored the importance of cultivating a blame-free patient safety culture among clinical managers and promoting reporting, sharing, and learning from mistakes.

Our study revealed associations between gender, hospital nature, and time delays per shift with perceived patient safety culture. The results align with those of a narrative synthesis of qualitative studies, which similarly highlighted that patient safety culture is influenced by staffing, organizational, and patient-related factors [46]. However, these findings diverged from a previous study showing that age and hospital level were positively associated with the attitudes of clinical nurse managers [27]. Additionally, we identified significant factors influencing patient safety grades and the number of reported events, which aligned with a previous study indicating the influence of working years in the hospital and working hours per week on patient safety grades and event reporting in surgical units, respectively [47]. Furthermore, our study demonstrated that a higher perceived patient safety culture positively correlated with improved patient safety grades, specifically in terms of the “teamwork within units”, “management support for patient safety”, “communication openness”, and “staffing” dimensions. These results were consistent with previous studies indicating that promoting a perceived patient safety culture leads to enhanced patient safety grades in hospitals [26, 32]. Likewise, other studies suggested that an improved patient safety culture contributes to increased reporting rates of adverse events in hospitals, particularly in dimensions related to “communication openness” and “handoffs & and transitions”, as supported by additional studies [48, 49].

This study represented the first investigation into factors associated with perceived patient safety culture among hospital clinical managers. The inclusion of clinical managers in hospitals addressed the research gap in this study, which played a pivotal role in managing patient safety culture. The findings support the further clinical development of patient safety culture among managers and offer practical suggestions for hospital management.

Nevertheless, several limitations warrant caution in interpreting the findings. First, the cross-sectional study design introduced potential bias. Future longitudinal studies are needed to explore causal relationships between patient safety culture and its contributing factors. Second, the samples were drawn from four hospitals in Changsha City, which may not fully represent clinical managers from other hospitals in different regions. Future multicenter studies at a national level are necessary to obtain a more representative sample. Third, the results regarding perceived patient safety culture may be subject to bias because of the self-reported data in the questionnaire survey. Future studies could benefit from incorporating additional methods, such as observations and interviews, to obtain more objective evaluations.

## Conclusion

The study identified three areas in perceived patient safety culture among clinical managers that require improvement: nonpunitive response to errors, staffing, and frequency of events reported. It is essential to establish a nonpunitive environment to promote reporting adverse events and facilitate organizational learning. Furthermore, there is a need to intensify efforts to effectively allocate staff resources to ensure patient safety. Additionally, expanding training programs on adverse event reporting to include clinical managers in hospitals is warranted. Finally, our findings emphasize the significance of participant characteristics in shaping their perceptions of patient safety culture and offer implications for future studies to develop targeted interventions based on these characteristics. More studies will be conducted to assess the feasibility and effectiveness of evidence-based proactive projects aimed at integrating patient safety culture into healthcare systems.

### Electronic supplementary material

Below is the link to the electronic supplementary material.

Supplementary Material 1

## Data Availability

All data generated or analyzed during this study are included in this published article. The datasets used and/or analyzed during the current study are available from the corresponding author upon reasonable request.

## Abbreviations

HSOPSC: Hospital Survey on Patient Safety Culture
PRR: Positive Response Rate
M: Mean
SD: Standard deviation
β: Beta coefficient
