# Supplementary material for: Perceived patient safety culture and its associated factors among clinical managers of tertiary hospitals: a cross-sectional survey
Source: BMC Nurs. 2023 Sep 25;22:329. doi: 10.1186/s12912-023-01494-4 (PMC10518958; doi:10.1186/s12912-023-01494-4)
Supplement: Supplementary file 1 — Supplementary Material 1 [file 12912_2023_1494_MOESM1_ESM.docx]

**Supplementary Table 1 The mean scores and PRRs for each item of the HSOPSC**

| **Items** | **Mean(SD)** | **PRRs^a^** | **PRRs^b^** |
| --- | --- | --- | --- |
| 1. Teamwork Within Units | 86.6(11.7) | 90.17 | 91 |
| A1. People support one another in this unit. | 88.8(14.2) | 91.34 | 94 |
| A3. When a lot of work needs to be done quickly, we work together as a team to get the work done | 86.6(14.0) | 89.35 | 94 |
| A4. In this unit, people treat each other with respect | 86.8(14.6) | 89.17 | 91 |
| A11. When one area in this unit gets really busy, others help out. | 83.4(14.2) | 87.36 | 83 |
| 2. Supervisor/Manager Expectations & Actions Promoting Patient Safety | 78.4(12.2) | 75.70 | 91 |
| B1. My supervisor/manager says a good word when he/she sees a job done according to established patient safety procedures | 82.4(14.4) | 83.39 | 90 |
| B2. My supervisor/manager seriously considers staff suggestions for improving patient safety | 83.8(13.4) | 86.64 | 92 |
| B3. Whenever pressure builds up, my supervisor/manager wants us to work faster, even if it means taking shortcuts. (Negatively worded) | 66.4(21.4) | 49.28 | 90 |
| B4. My supervisor/manager overlooks patient safety problems that happen over and over. (Negatively worded) | 78.8(18.8) | 78.16 | 91 |
| 3. Organizational Learning—Continuous Improvement | 84.4(10.2) | 89.55 | 85 |
| A6. We are actively doing things to improve patient safety | 89.2(12.8) | 94.40 | 90 |
| A9. Mistakes have led to positive changes here. | 80.4(14.0) | 83.21 | 82 |
| A13. After we make changes to improve patient safety, we evaluate their effectiveness. | 82.8(13.4) | 88.27 | 83 |
| 4. Management Support for Patient Safety | 77.5(13.8) | 71.43 | 85 |
| F1. Hospital management provides a work climate that promotes patient safety. | 79.6(15.8) | 77.44 | 92 |
| F8. The actions of hospital management show that patient safety is a top priority. | 81.6(16.4) | 77.98 | 88 |
| F9. Hospital management seems interested in patient safety only after an adverse event happens. (Negatively worded) | 70.4(19.0) | 56.68 | 75 |
| 5. Overall Perceptions of Patient Safety | 73.2(12.5) | 62.29 | 79 |
| A15. Patient safety is never sacrificed to get more work done | 83.6(17.8) | 83.21 | 77 |
| A18. Our procedures and systems are good at preventing errors from happening. | 74.6(15.8) | 65.34 | 84 |
| A10. It is just by chance that more serious mistakes don't happen around here. (Negatively worded) | 72.2(20.2) | 61.01 | 77 |
| A17. We have patient safety problems in this unit. (Negatively worded) | 61.0(20.8) | 65.34 | 78 |
| 6. Feedback & Communication About Error | 80.2(12.2) | 77.61 | 83 |
| C1. We are given feedback about changes put into place based on event reports. | 80.6(15.6) | 79.24 | 75 |
| C3. We are informed about errors that happen in this unit. | 74.6(18.0) | 62.82 | 83 |
| C5. In this unit, we discuss ways to prevent errors from happening again. | 85.2(13.4) | 88.99 | 90 |
| 7. Communication Openness | 71.6(12.4) | 57.58 | 82 |
| C2. Staff will freely speak up if they see something that may negatively affect patient care | 80.4(14.8) | 78.34 | 89 |
| C4. Staff feels free to question the decisions or actions of those with more authority. | 63.0(19.8) | 34.48 | 74 |
| C6. Staff are afraid to ask questions when something does not seem right. (Negatively worded | 71.0(19.0) | 57.76 | 82 |
| 8. Frequency of Events Reported | 68.3(16.9) | 47.37 | 76 |
| D1. When a mistake is made, but is caught and corrected before affecting the patient, how often is this reported? | 71.0(19.2) | 53.61 | 71 |
| D2. When a mistake is made, but has no potential to harm the patient, how often is this reported? | 65.8(20.0) | 40.79 | 71 |
| D3. When a mistake is made that could harm the patient, but does not, how often is this reported? | 68.8(20.6) | 48.19 | 85 |
| 9. Teamwork Across Units | 72.4(12.0) | 59.88 | 72 |
| F4. There is good cooperation among hospital units that need to work together. | 77.8(14.0) | 72.2 | 73 |
| F10. Hospital units work well together to provide the best care for patients. | 76.8(15.4) | 67.33 | 84 |
| F2. Hospital units do not coordinate well with each other. (Negatively worded) | 62.4(19.4) | 37.18 | 77 |
| F6. It is often unpleasant to work with staff from other hospital units. (Negatively worded) | 71.6(16.2) | 59.39 | 78 |
| 10. Staffing | 63.5(14.8) | 41.88 | 65 |
| A2. We have enough staff to handle the workload. | 66.8(22.0) | 44.95 | 67 |
| A5. Staff in this unit work longer hours than is best for patient care. (Negatively worded) | 56.6(23.0) | 30.32 | 58 |
| A7. We use more agency/temporary staff than is best for patient care. (Negatively worded) | 72.2(21.0) | 61.01 | 71 |
| A14. We work in "crisis mode" trying to do too much, too quickly. (Negatively worded) | 57.2(20.4) | 28.16 | 63 |
| 11. Handoffs & Transitions | 68.6(13.2) | 52.92 | 52 |
| F3. Things "fall between the cracks" when transferring patients from one unit to another. (Negatively worded) | 64.4(19.2) | 89.35 | 48 |
| F5. Important patient care information is often lost during shift changes. (Negatively worded) | 75.6(16.2) | 30.32 | 56 |
| F7. Problems often occur in the exchange of information across hospital units. * | 71.2(16.2) | 61.01 | 53 |
| F11. Shift changes are problematic for patients in this hospital. * | 61.0(20.0) | 87.36 | 52 |
| 12. Non-punitive Response to Errors | 62.1(14.9) | 40.51 | 70 |
| A8. Staff feels like their mistakes are held against them. * | 66.6(21.2) | 46.21 | 75 |
| A12. When an event is reported, it feels like the person is being written up, not the problem. * | 70.6(20.2) | 59.21 | 76 |
| A16. Staff worry that mistakes they make are kept in their personnel file. * | 47.4(19.0) | 12.82 | 58 |

HSOPSC, Hospital Survey on Patient Safety Culture; PRRs, Positive Response Rates.

* indicates a negatively worded item.

^a^ Positive response rates (%) of each item and dimension among hospital managers in China

^b^ Positive response rates (%) of managers in the USA according to the 2021 SOPS Hospital 1.0 Database

Changsha

Randomly select 4 hospitals

Administrate questionnaires

Managers: N=560

Changsha

Randomly select 2 out of 4 areas

Hospital 1

N=143

Hospital 2

N=134

Hospital 3

N=247

Hospital 4

N=36

**Supplementary Figure 1 Sample Frame**

Returned

N=141 (98.6%)

Returned

N=132 (98.5%)

Returned

N=246

(99.6%)

Returned

N=35

(97.2%)

The total questionnaires

N=539 (96.3%)
